# Ethiopian medicinal plants used for their anti-inflammatory, wound healing or anti-infective activities: protocol for systematic literature review and meta-analysis

**DOI:** 10.1136/bmjos-2020-100064

**Published:** 2020-09-03

**Authors:** Dereje Nigussie, Belete Adefris Legesse, Gail Davey, Abebaw Fekadu, Eyasu Makonnen

**Affiliations:** 1 Addis Ababa University, College of Health Sciences, Centre for Innovative Drug Development and Therapeutic Trials for Africa (CDT-Africa), P.O. Box: 9086, Addis Ababa, Ethiopia, Addis Ababa, Ethiopia; 2 Centre for Global Health Research, Brighton and Sussex Medical School, University of Sussex, Brighton, BN1 9PX, UK, Brighton, UK; 3 School of Public Health, Addis Ababa University, Addis Ababa, Ethiopia; 4 Department of Pharmacology and Clinical Pharmacy, College of Health Sciences, Addis Ababa University, Addis Ababa, Ethiopia

## Abstract

**Objectives:**

Medicinal plants are used globally as alternative medicines in the management of a range of disease conditions and are widely accepted across differing societies. Ethiopia hosts a large number of plant species (>7000 higher plant species), of which around 12% are thought to be endemic, making it a rich source of plant extracts potentially useful for human health. The aim of this review is to evaluate Ethiopian medicinal plants for their anti-inflammatory, wound healing, antifungal or antibacterial activities.

**Methods and analysis:**

The guidance of the Preferred Reporting Items for Systematic Review and Meta-analysis Protocols (PRISMA-P) statement will be used. This review will consider all controlled studies of anti-inflammatory and wound healing properties (both in vivo and in vitro) and in vitro anti-infective properties of medicinal plants found in Ethiopia. Data sources will be EMBASE, PubMed/Medline, Scopus and Google Scholar. Guidance documents on good in vitro methods and checklists for reporting in vitro studies will be used for quality assessment of in vitro studies. The risk of bias tool for animal intervention studies (the SYRCLE RoB tool) will be used to assess the validity of studies. The main outcomes will be percent inhibition of inflammation, time of epithelisation and tissue tensile strength in wounds and microbial growth inhibition.

**Ethics and dissemination:**

The findings of this systematic review will be disseminated by publishing in a peer-reviewed journal and via conference presentations. Ethical clearance was obtained from the Brighton and Sussex Medical School, Research Governance & Ethics Committee (RGEC) and Addis Ababa University, College of Health Science, Institutional Review Board.

**PROSPERO registration number:**

This systematic literature review has been registered with PROSPERO (registration number CRD42019127471).

**Strengths and limitations of this study**

- This is the first systematic review to assess Ethiopian medicinal plants for inflammation, wound healing and anti-infection properties.
- The review includes both in vivo and in vitro studies.
- Study screening and data extraction will be conducted independently by two authors.
- This systematic review only considers studies written in English.
- It will consider a wide range of methodological approaches and uses of different types of interventions which may result in heterogeneous data. As a result of this heterogeneity, it may be impossible to conduct meta-analysis.

## Introduction

Herbal medicines are used worldwide as alternative treatments for a range of conditions and are widely accepted across a range of cultures and settings. Their medicinal values arise from the active substances produced by the plants as secondary metabolites, which themselves produce physiological changes in the human body.^1^ Most of the drugs currently used against infectious agents are derived from natural products or from structures suggested by natural product ‘leads’—chemical compounds with pharmacological or biological activity likely to be therapeutically useful, but which require modification to fit better to the target.^2^

In the search for improved, safe, effective and affordable drugs for treatment of tropical lymphoedema, plant-derived products represent an attractive option.^3^ Herbal products are relatively safe, chemically complex mixtures composed of a range of constituents with multiple potential targets and different mechanisms of action.^4^ Phytochemicals found in certain herbal extracts are reported to demonstrate analgesic and anti-inflammatory properties.^1^ Most of these act in a comparable manner to non-steroidal anti-inflammatory drugs on inflammatory pathways.^5^ Similarly, evidence suggests that medicinal plant products may be cost-effective promoters of wound healing.^6^

Through its geographical position, range of altitude, rainfall pattern and soil variability, Ethiopia has immense ecological diversity. Ethiopia hosts a large number of plant species (>7000 higher plant species), of which around 12% are thought to be endemic, making it a rich source of plant extracts potentially useful for human health.^7^ In Ethiopia, traditional medicine is culturally entrenched in all communities, and many Ethiopian medicinal plants are claimed to have anti-inflammatory and wound healing activities.^8^ Phytochemical and pharmacological investigations of endemic plant extracts for the care of wounds and swelling caused by bacterial infections have shown anti-inflammatory and diuretic activities.^9 10^

One of the most neglected health issues within Ethiopia is tropical lymphoedema. Over 1.5 million Ethiopians are estimated to be affected by lymphoedema, predominantly caused by podoconiosis.^11^ Lymphoedema is a condition caused by failure of lymphatic drainage leading to accumulation of protein-rich fluid in the interstitial spaces. Primary lymphoedema arises from genetic disorders, while secondary lymphoedema arises from damage to the lymphatic system, due to lymphatic vessel infestation, mineral damage, recurrent infection, lymphadenectomy or radiotherapy in patients with cancer.^12 13^

The aims of treatment of lymphoedema are to improve lymph drainage and reduce the incidence of ‘acute attacks’ (acute dermatolymphangioadenitis). Acute attacks are characterised by local inflammation of the skin, lymph nodes and lymphatic vessels resulting in very high fever, confusion, headache, rigours, chills and pain of the lymph glands.^14^ Although foot hygiene and skin care has recently been shown to be a resource-frugal approach to reducing frequency of acute attacks,^15^ few pharmacological approaches exist. This review therefore focuses on preclinical studies of Ethiopian medicinal plants used to manage inflammation, wound and infection.

Preclinical studies include animal and in vitro studies which elucidate mechanisms of disease at the molecular level and may be used for drug screening before testing on humans. Many in vitro and in vivo studies have been conducted on the safety of Ethiopian medicinal plants and their efficacy against inflammation, infection and for wound healing. However, data from these studies have never been compiled. This systematic review aims to draw together information on Ethiopian medicinal plants used as anti-inflammatory, wound healing and anti-infective agents that might potentially be used for treatment of lymphoedema and associated wounds. We intend to answer the following questions:

- In in vitro and in vivo (animal) studies, do Ethiopian medicinal plants have anti-infective, anti-inflammatory or wound healing activities compared with conventional treatments or placebo?
- Which secondary metabolites of medicinal plants found in Ethiopia have been investigated for anti-infective, anti-inflammatory or wound healing activities?
- What experimental models are most frequently used to investigate the efficacy of medicinal plants and their compounds?

In the context of this review, ‘Ethiopian medicinal plants’ is defined as follows:

*‘Ethiopian medicinal plants’* refers to plants which are found in Ethiopia and have been used traditionally for medicinal purposes by societies in Ethiopia and elsewhere.

## Methods and analysis

This systematic review protocol uses the guidance of the Preferred Reporting Items for Systematic Review and Meta-analysis Protocols (PRISMA-P) statement, and a completed PRISMA-P checklist has been included with this submission.^16^ This systematic review is registered in PROSPERO and the registration number is CRD42019127471.

### Study design

This review considers all controlled in vivo and in vitro studies conducted on anti-inflammatory activity and wound healing, and in vitro anti-infective studies evaluating the efficacy and safety of Ethiopian medicinal plants.

The components population (disease model), exposure (intervention), comparator and outcome (PICO) of this review will be as follows:

#### Disease model

This includes laboratory animals (healthy mice and rats) used after experimentally induced inflammation and wound scratches; and/or cell lines used in in vitro models for anti-inflammatory and wound healing assays. Similarly, micro-organisms (bacteria and fungi) which were used to assess anti-infective activity.

##### Intervention

This includes extracts, fractions and/or compounds from different parts of the plants such seeds, roots, flowers, buds and leaves used in the experimental (test) groups; and conventional drugs and placebo used in control groups. Medicinal plants used will be regardless of their method of preparations (maceration, decoctions, Soxhlet, steam distillation methods), but not synthesised compounds. There is no restriction on dosage form, concentration, frequency of administration (treatment), dose, duration of medicinal plants exposure and time of measurement of outcomes.

##### Comparator

This includes placebo, vehicle and/or conventional (reference) drugs used for treatment of controls. Conventional (reference) drugs are known anti-inflammatory, wound healing or anti-infective agents which are known to produce results similar to those predicted by the hypothesis. They are used as a benchmark against which to predict the efficacy and safety of plant substances. Vehicles are not expected to produce an effect but help to identify outside influences on the experiment, such as contamination. Similarly, for placebo (non-intervention) controls, groups will not receive a treatment or given an inert substance.

##### Outcomes

The primary outcomes that will be analysed in this review will be as follows:

For in vivo anti-inflammatory studies, linear paw circumference using a plethysmometer or water displacement method to measure the volume of oedematous legs.

For in vitro studies, percent inhibition of cyclo-oxygenase enzyme production, inflammatory biomarkers such as vascular cell adhesion molecule-1, interferon gamma (IFN-γ)-induced protein 10, monokine-induced by interferon, nitric oxide (NO) production, interleukin-1β (IL-1β) and IL-6 mRNA expressions, quantity of proinflammatory cytokines (tumour necrosis factor-α (TNF-α) and IL-6), median inhibitory concentration (IC_50_) values, percentage inhibition of denatured protein and gene expression of inflammatory cells using molecular techniques such as ELISA and western blot, and NO assay by measuring optical density, and measuring the amount of gene expressed.

For in vivo wound healing, wound contraction (area of wound), percent tensile wound strength (skin breaking strength) using water flow method, period of epithelisation (the number of days required for falling off the dead tissue), collagen formation, fibroblast proliferation and angiogenesis evaluated microscopically.

For in vitro wound healing studies, cell proliferation and migration rates measured using UV spectrophotometer and calculated as percent cell viability, and IC_50_ for free radical scavenging activity of medicinal plants.

For the anti-infective studies, diameter (mm) of the zone of inhibition of bacterial growth measured at each test level for agar well and paper disc diffusion assay. For the microdilution methods, minimum inhibitory concentration (MIC) and minimum bactericidal concentration (MBC) visually identified as colour changes (colorimetric methods), or clear or turbid solutions (non-colorimetric methods).

#### Eligibility criteria

Inclusion criteria:

- Published works including theses, articles and proceedings, which deal with efficacy evaluation of antibacterial, antifungal, anti-inflammatory and wound healing activities in in vivo and in vitro studies.

Exclusion criteria:

- Newspaper articles.
- Unpublished work.
- Reviews.

### Information sources

We will conduct searches in electronic databases using a combination of free text keywords and Medical Subject Heading (MeSH) terms. EMBASE, PUBMED/MEDLINE, Scopus of Science and Google Scholar will be used as sources of information for the search. Grey literature such as theses and dissertations, technical reports, working papers, evaluation reports, conference proceedings, patents and preprints will be included in the review.

### Search strategy

The search will include all articles containing the descriptors published up to 30 August 2019 for all databases. There will be restriction of language to English for the identified articles in all databases. The search strategy will include all articles containing the descriptors. Structured search strategies will be developed using the vocabulary terms of each database and targeting the ‘title’ and ‘abstract’ fields. We will also search manually using the references of previously published works. The following search terms will be used: Ethiopia, medicinal plants, Ethiopian medicinal plants, herbal products, care, management, therapeutic, lymphoedema, lymphedema, swelling, podoconiosis, elephantiasis, wound, wound healing, inflammation, anti-inflammatory, bacteria, antibacterial, fungi, anti-infective, antimicrobial, anti-fungal and other related words or phrases. A PUBMED search is included in online supplementary annex 1.

10.1136/bmjos-2020-100064.supp1 Supplementary data

### Selection of studies

After electronic searching, the records will be uploaded to Mendeley. Assessors will pilot some studies before undertaking full study selection. All studies will be screened independently by two investigators (DN and BL) by scanning the titles and abstracts of the articles based on the inclusion criteria. For the documents that fit the inclusion criteria, the investigators will read the entire article to confirm if it meets the criteria and prepare to extract relevant information. Disagreements will be resolved by discussion between the two investigators and if disagreement persists, we will discuss with a third party (GD).

### Data extraction

Two reviewers (DN and BL) will independently extract data using a data extraction form and summarise experimental works including study types. The Cochrane data collection form for interventional reviews will be customised to our situation and used for data extraction. A calibration exercise will be done before starting the review to ensure consistency across the reviewers. Reviewers will resolve disagreements with the help of the third investigator (GD or EM). Authors will be contacted if information is unclear. The following data will be extracted from the respective study models.

For the animal studies: title of the study, name of the first author, year of publication, type of study, country where study conducted, species/(sub/strain) of laboratory animals, number of groups and number of animals per group, types of diet used for laboratory animals, housing condition of laboratory animals, scientific name of the medicinal plant(s), vernacular name of medicinal plants, family of the medicinal plants, voucher number, whether plants identified by a botanist/herbalist, plant parts used/extracted, types of extracts/fractions/compounds used, positive controls used, assay methods (model) used, dose regimen (dose level or concentration of plant material used per group and frequency), route of administration, duration of the exposure of animals to treatment, time of measurement/observation period of outcomes, endpoints measured, statistical method used, dose or concentration (eg, mean, median, frequency, measures of precision or variance) used, statistical significance of dose levels, author’s interpretation and outcome measured at a population level or individual level.

For the in vitro studies: title of the study, name of the first author, year of publication, health outcome category, potential conflict of interest, country where the study conducted, type of study, name of the assay, study design, aim of the study, name and sources of cell lines/kits, name and source of media used, disease model, scientific name of the plant(s)/compounds, vernacular name, family of the plants, plant authenticated/identified, voucher number, plant parts used/extracted, extraction method used, types of plant extracts/fractions/compounds, negative control used, positive controls used, concentration regimen (dose level or concentration of plant material used per group and frequency), duration of the exposure, number of test groups/number of cell line per group, how many experimental duplicates were conducted, time of measurement/observation period, endpoints measured, methods to measure endpoint, are study designs clearly stated, statistical method used, results per dose or concentration (eg, mean, median, frequency, measures of precision or variance), statistical significance of other dose levels, author's interpretation, outcome measured at a population level or individual level and whether outcome measures meet the criteria for inclusion.

When individual studies have multiple treatment groups, we will combine the groups from multiple arms into one group to avoid the possibility of introducing bias caused by multiple statistical comparisons with one control group.^16^

### Outcome measured

For the in vivo studies of anti-inflammatory activity, the primary outcomes will be percent inhibition of the carrageenan-induced oedema and/or the percent inhibition of the weight of granuloma tissue formation relative to the controls. In in vitro anti-inflammatory studies, the primary outcomes will be percent inhibition of inflammatory cells and proinflammatory cells which includes percent inhibition of lipoxygenase enzymes, percent inhibition of protein denaturation, levels of inflammatory cytokines (TNF-α, IL-6, IL-10 and IFN-γ), level of cyclo-oxygenase and concentration of NO in inflammation-induced cell lines after treatment by plant extracts. The type of data (variables) that will be extracted for anti-inflammatory activity, wound healing and anti-infective activity are continuous (percentage and µg/mL, mg/mL).

In in vivo wound healing assays, the primary outcomes will be percentage wound contraction, period of epithelisation and percent of wound tensile strength in experimentally induced wounds in laboratory animals, whereas for the in vitro wound healing assays, relative cell spreading and migration, percent cell proliferation and viability, and free radical scavenging activity of medicinal plants will be the primary outcomes. In in vitro studies of anti-infective activity, percent inhibition of growth of micro-organisms, MIC and concentration that inhibits 50% of the growth of micro-organisms (IC_50_) will be the primary outcomes. Secondary outcomes will be long-term toxicity, death of animals and experiment dropouts.

### Assessment of risk of bias

Two review authors (DN and BL) will independently assess the risk of bias for each study included. Disagreements will be resolved by consensus or by consulting a third party (GD or EM). The critical appraisal process for in vivo anti-inflammatory activity and wound healing will be performed using the Risk of Bias tool for animal intervention studies (SYRCLE’s RoB tool)^17^ and Animal Research: Reporting of In vivo Experiments (ARRIVE) guidelines to assess the internal validity of the studies.^18^ These tools will be used to assess the methodological quality of studies to generate reliable information and create transparency. Studies with highly unacceptable levels of bias will be excluded. A highly unacceptable level of bias occurs when studies have serious errors in conducting, analysis or reporting, have large amounts of missing information or discrepancies in reporting. For instance, in randomisation of experimental animals, if there is direct evidence that animals were allocated to study groups using a non-random method, the study would be categorised as ‘definitely high risk of bias’. The judgement of bias will be categorised as yes, no or unclear. A ‘yes’ judgement indicates a low risk of bias; a ‘no’ judgement indicates high risk of bias; the judgement will be ‘unclear’ if insufficient details are reported to assess the risk of bias properly. Studies will be evaluated for their internal and external validity. Reviewers will judge the risk of bias for individual elements from five domains of bias (selection, performance, attrition, reporting and other) using the SYRCLE’s risk of bias tool for animal studies—Appendix D—and decide the inclusion and exclusion of the studies.^17^

The following criteria will be used to assess the quality of individual in vivo studies:

- Systematic differences between study groups at the start of an experiment (selection bias).
  - Did the investigators describe a random component in the sequence generation process (eg, methods used for randomisation of the animals)?
  - Balanced distribution of relevant baseline characteristics for the intervention and control groups (eg, age, sex, weight of the animals).
  - If relevant, did the investigators adequately adjust for unequal distribution of some relevant baseline characteristics in the analysis?
- Systematic differences occur in how the groups are handled during a study (performance bias).
  - Adequacy of timing of disease induction in both the test and the control groups.
  - Experimental animals random housing to test and control group, and feeding conditions.
  - Was the allocation to the different groups adequately concealed during the study such as blinding of the caregivers and/or investigators from knowing which intervention each animal received during the experiment (labelling the cages and drug containers with code)?
- Circumstances during the experiment in both experimental and control groups.
  - Timing of administration of the placebo and experimental extracts.
  - Instruments used to conduct experiment differ between experimental and control groups.
- Systematic differences occur between groups in how outcomes are ascertained, diagnosed or verified (detection bias).
  - Was the outcome assessor blinded? If not blinded, do review authors judge that the outcome is not likely to be influenced by lack of blinding?
  - Did the investigators randomly pick an animal during outcome assessment, or did they use a random component in the sequence generation for outcome assessment?
- Whether all animals receiving the same intervention are caged together, but analysis was conducted as if every single animal was one experimental unit.
- Incomplete data (attrition bias).
  - Were all animals included in the analysis?
  - Are missing outcome data imputed using appropriate methods?
  - Are missing outcome data balanced in numbers across intervention groups, with similar reasons for missing data across groups?
- Selective reporting (reporting bias).
  - Was the study protocol available and were all of the study’s prespecified primary and secondary outcomes reported in the current manuscript?
  - Was the study protocol not available, but was it clear that the published report included all expected outcomes (ie, comparing the Methods and Results sections).
- Other biases—unit of analysis errors, inappropriate influence of funders and adding new laboratory animals to replace dropouts from the original population and so on.

The critical appraisal process for in vitro anti-inflammatory activity and wound healing will be performed using the Guidance Document on Good In vitro Method Practices (GIVIMP)^19^ and the Checklist for Reporting In vitro Studies guidelines.^20^ For the in vitro antibacterial studies, checklists for good practice for pharmaceutical microbiology laboratories (WHO) will be customised and used for quality assessment.^21 22^ The following key criteria will be used to assess the quality of individual in vitro studies:

- Assurance of the quality of all materials and methods, and of their use and application, in order to maintain the integrity, validity and reproducibility of the laboratory work conducted.
  - Test definition (including purpose, need and scientific basis).
  - Laboratory reproducibility, validity and international acceptance of the in vitro method(s).
  - Clearly written and well-documented in vitro method description, and related standard operating procedure (SOP).
  - Did the in vitro method(s) include all relevant and reliable positive and negative controls, including acceptance criteria?
  - SOP/guidelines cell culture maintenance, and safety practices for use and disposal of the test system, including transport and containments.
  - Relevant documentation of proof of sterility, date of arrival, expiry dates and batch numbers (as the suitability and acceptability) of laboratory consumables (materials).
  - Evidence of provision of relevant and adequate education and training for all personnel, to promote high quality work and safety.
- Are the in vitro cell and tissue culture facilities fit for purpose? Evidence of quality laboratory management maintained:
  - Was the facility designed or adapted to minimise the risk of errors (eg, mix-ups) and to avoid (cross-contamination) which may adversely affect the quality of the work performed?
  - Is appropriate environment maintained for the type of work conducted in the laboratory (appropriate biosafety level)?
  - Is there an appropriate documented procedure for disinfection of work surfaces, safety cabinets and equipment?
  - Any establishment and maintenance of adequate measures to protect individuals and the environment from any potential hazards.
- Is equipment regularly maintained, monitored and calibrated?
- Compliance of laboratory suppliers with good laboratory practice principles, such that test system providers should adhere to a formal quality system, such as International Standards (Good Manufacturing Practice, Good Laboratory Practices, International Organization for Standards).
- Evidence that the cell lines are free from any contaminants, indicate functionality, genetic stability and identity; reference data to assess the relevance of in vitro methods; does the media and serum used precisely specified (source, batch number, expiry date, components) and meet the required specifications; does the maximum acceptable levels of serum components, such as immunoglobulins and haemoglobin are defined well.
- Are reference and control items described well, such as negative and positive controls? Suitability of reference and control items and justification for the selection of the reference item(s), stability and solubility of the reference and control items.
- Are applicability domain of the in vitro method described well, as well as any limitations or exceptions?
- Is concentration of solvent(s) used without interfering with the in vitro method? Compatibility and toxicity of the solvent with the test system assessed, to select the appropriate solvent at an acceptable final concentration in the in vitro method medium.
- Is the number of replicates for each testing condition, including concentration level(s) used for the reference and control item(s), and test items and so on, specified?
- Is there evidence that cell seeding, treatment and measurement is performed in a uniform fashion across the whole plate (well-to-well), between plates and across multiple runs (minimise any potential systematic effects).
- Statistical method used for data analysis and interpretation.

Then, risk of bias criteria will be judged as ‘low’, ‘high’ or ‘unclear’. We plan to report only studies in which risk of bias is low or moderate and to omit high risk of bias studies from analysis.

### Data synthesis

All included studies will be classified for data synthesis into six different models according to the type and purpose of the studies. These are in vivo and in vitro anti-inflammatory studies, in vivo and in vitro wound healing studies, antibacterial and anti-fungal activity studies. For the narrative synthesis, methods and techniques such as textual description of studies, groupings, tabulation, thematic and content analysis for translating data will be used. Then, a narrative (qualitative) synthesis which describes the characteristics of studies and compares the effect of each plant extract relative to control, main parameters measured/analysed, quality of included studies and the risk of bias of all studies will be described and data will be presented in a table.

#### Assessment of heterogeneity

We will evaluate heterogeneity descriptively from the narrative synthesised data, and potential reasons for heterogeneity will be found by examining individual study and subgroup characteristics. From the qualitative review, similarity in intervention, methodology and statistical methods used among the studies will be considered good evidence for homogeneity. In the case of interventional, methodological or statistical heterogeneity, study results will not be reported as a pooled effect estimate in meta-analysis. In addition, if meta-analysis is possible, statistical heterogeneity will be also tested using the χ^2^ test (significance level: 0.1) and the I^2^ statistic. If statistical heterogeneity is observed (I^2^>=50% or p<0.1), the random effects model will be used.

The Mantel-Haenszel method will be used for the fixed effect model if tests of heterogeneity are not significant. If heterogeneity is substantial, we will not perform a meta-analysis; a narrative, qualitative summary will be done. If there is no good evidence for homogeneous effects across studies of low risk bias, data will be summarised by means of a random-effects model^23^; and random-effects meta-analyses will be interpreted with consideration of the whole distribution effects by presenting a prediction interval.^23^ Statistical analysis will be performed according to statistical guidelines of the *Cochrane Handbook for Systematic Reviews of Interventions*.^24^

#### Measurement of treatment effect

Data will be analysed by weighted mean differences with 95% CI or standardised mean differences if different measurement scales are used. Skewed data and non-quantitative data will be presented descriptively.

#### Unit of analysis

The primary analysis will include individually randomised studies. All included studies will be assessed to determine the unit of randomisation and whether this unit of randomisation is consistent with the unit of analysis. For cluster randomised trials, interclass correlation coefficients will be extracted to modify the results using the methods described in *The Cochrane Handbook for Systematic Reviews of Interventions*. In studies of more than two treatment groups, additional treatment arms will be presented. Where the additional treatment arms are not relevant, they will not be considered. In this systematic review outcomes will be measured with percent and µg/mL, mg/mL.

#### Dealing with missing data

If there are missing data, the original author of the study will be contacted. If missing data cannot be obtained or if SD for outcomes are not complete, these values will be imputed by assuming the SD of the missing outcome to be the average of the SD from those studies where this information was reported; and impact of imputation on meta-analyses will be assessed by means of sensitivity analysis.

#### Sensitivity analysis

To determine publication bias, we will examine whether the protocol was published before the animal or in vitro study was executed. The potential for reporting bias will be further explored by funnel plot and Egger’s regression test for 10 and more studies. An asymmetrical funnel plot or a p value of <0.10 on Egger’s test will be considered to indicate the presence of publication bias. For very small sample size, the pooled SD will be measured in the formula for precision (1/variance) in Egger regression. Selective reporting of outcomes will be also evaluated (outcome reporting bias). Small sample bias will also be assessed by comparing fixed estimated effect with the random-effects model. An option of comparing outcomes reported in the Methods and Results sections will be used when a protocol is not available.

### Assessing certainty of evidence included in the review

Grading of the systematic review will be done using the guidelines of the Grading of Recommendations, Assessment, Development and Evaluation (GRADE) system which has been adapted for preclinical animal interventional studies. Quality of evidence will be categorised as high, moderate, low or very low quality.^25^

## Discussion

Systematic reviews provide the highest quality evidence on effectiveness of a treatment or service. By conducting this review, we will generate evidence for potential intervention compounds derived from Ethiopian medicinal plants that may be of value to explore more thoroughly for treatment of tropical lymphoedema.
