# Supplementary material for: Ethiopian medicinal plants used for their anti-inflammatory, wound healing or anti-infective activities: protocol for systematic literature review and meta-analysis
Source: BMJ Open Sci. 2020 Sep 3;4(1):e100064. doi: 10.1136/bmjos-2020-100064 (PMC8647601; doi:10.1136/bmjos-2020-100064)
Supplement: Supplementary data [file bmjos-2020-100064supp001.pdf]

Annex 1

Title: Ethiopian medicinal plants used for their anti-inflammatory, wound healing or anti-infective activities - Protocol for systematic literature review and meta-analysis

PubMed searching strategy

Logic grid

	Population	Intervention	Comparator	Outcome
	Experimental animals and/or cell lines	Treatment with plant extracts	Convention-al drugs	Reduced inflammation, wound healing, microbial growth inhibition
Search terms: key words	Laboratory animals [tw] OR *in vivo* [tw] OR "experimental animals" [tw] OR "Cell lines" [tw] OR *in vitro* [tw]	Treatment [tw] OR management [tw] OR therapy [tw] OR care [tw]	Anti-inflammatory, anti-bacterial drugs	Anti-inflammatory [tw] OR Anti-bacterial [tw] OR ''wound healing'' [tw]

S/N	Query	Search Results
1	**[tw] – text words:**<br>"Animal model" [tw] OR "disease model" [tw] OR "experimental animals" [tw] OR "laboratory animals" [tw] OR *in vivo* [tw] "*in vivo* method" [tw] OR *in vitro* [tw] OR "*in vitro* method" [tw] OR "cell lines" [tw]	**1,340,422**
2	**MeSH - *In vivo*:**<br>((((((((("beta-amyloid peptide (29-40)" [Supplementary Concept]) AND "dynorphin amide (1-13), Ala(2)-(5-F-Phe)(4)-" [Supplementary Concept]) AND "20-carboxy-N-acetylleukotriene E4" [Supplementary Concept]) AND "Ro 31-8830" [Supplementary Concept]) AND "LsaA protein, Lawsonia intracellularis" [Supplementary Concept]) AND "BMS279700" [Supplementary Concept]) AND "LASSBio-881" [Supplementary Concept]) AND "FR290581" [Supplementary Concept]) AND "Drug Screening Assays, Antitumor"[Mesh]) AND ("Drug Carriers/drug effects"[Mesh] OR "Drug Carriers/isolation and purification"[Mesh] OR "Drug Carriers/pharmacology"[Mesh] OR "Drug Carriers/therapeutic use"[Mesh] OR "Drug Carriers/toxicity"[Mesh])) AND ("Biological Assay/statistics and numerical data"[Mesh] OR "Biological Assay/therapeutic use"[Mesh])	**321**
**3**	**MeSH – "animals' model":**	**18**

	("Models, Animal"[Mesh] AND "Mice, Inbred SENCAR"[Mesh] AND "Mice, Inbred mdx"[Mesh])	
**4**	**laboratory animals** MeSH –<br>"Animals, Laboratory"[Mesh]	**14**
**5**	**MeSH - *In vitro:***<br>"*In Vitro* Techniques"[Mesh] AND "Diagnostic Test Approval"[Mesh]	**9**
**6**	**MeSH – "cell lines":**<br>"Cell Line"[Mesh] AND "Cell Line, Tumor"[Mesh] AND "PC12 Cells"[Mesh] AND "Cell Line, Transformed"[Mesh] AND "THP-1 Cells"[Mesh] AND "A549 Cells"[Mesh] AND "BALB 3T3 Cells"[Mesh] AND "Tumor Necrosis Factor-alpha"[Mesh] AND "marinoquinoline A" [Supplementary Concept] AND "3T3 Cells"[Mesh]	**165**
7	**[tw] – text words:**<br>"medicinal plants" [tw] OR "medicinal herbs" OR "herbal products" [tw] OR "natural products" [tw] OR "plant extracts" [tw] OR "phytochemicals" [tw] OR "secondary metabolites" [tw] OR "phytoconstituents" [tw]	**62332**
8	**MeSH – "Medicinal plants"**<br>"Medicine, African Traditional"[Mesh] AND "Drug Evaluation, Preclinical"[Mesh] AND "jigrine" [Supplementary Concept] AND "Plants, Medicinal"[Mesh]	**27**
9	**MeSH – "Plant extracts":**<br>"Plant Extracts"[Mesh] AND "Drugs, Chinese Herbal"[Mesh] AND "Phytoestrogens"[Mesh] AND "Ethnobotany"[Mesh] AND "Plants, Medicinal"[Mesh] AND "Phytotherapy"[Mesh] AND "Materia Medica"[Mesh]	**38**
10	**MeSH - "Secondary Metabolites":**<br>"Anthranilate Synthase"[Mesh] AND "myxochromide A3" [Supplementary Concept] AND "myxochromide B3" [Supplementary Concept] AND "knipholone-8-O-gentiobioside" [Supplementary Concept] AND "chrysalodin-10-gentiobioside" [Supplementary Concept] AND "tilivalline" [Supplementary Concept] AND "3,5-dibromotyrosine" [Supplementary Concept]	**7**
11	**[tw] – text words:**<br>Anti-inflammatory [tw] OR inflammation [tw] OR "inhibits inflammation" [tw] OR "inflammatory cytokines" [tw] OR "inflammatory cells" [tw] OR inhibits inflammatory cells [tw]	**601168**
12	**MeSH – Anti-inflammatory:**<br>("Anti-Inflammatory Agents"[Mesh] AND "Anti-Inflammatory Agents, Non-Steroidal"[Mesh] AND "Anti-Inflammatory Agents" [Pharmacological Action] AND "Anti-Inflammatory Agents, Non-Steroidal" [Pharmacological Action] AND "Mitogen-Activated Protein Kinase 14"[Mesh] AND "Inflammation"[Mesh] AND "Fever"[Mesh])	**24**

	AND ("Cytochrome P-450 CYP2C9"[Mesh] OR "ingramon" [Supplementary Concept] OR "Tolmetin"[Mesh] OR "Fenamates"[Mesh] OR "GDF15 protein, human" [Supplementary Concept] OR "5-(4-chlorobenzoyl)amino-N-(5-methyl-2-pyridyl)-3-methyl-4-isothiazolocarboxamide" [Supplementary Concept] OR "5-(4-chlorobenzoyl)amino-N-(4-chlorophenyl)-N-methyl-3-methyl-4-isothiazolecarboxamide" [Supplementary Concept] OR "VAF347" [Supplementary Concept] OR "5-(4-chlorobenzoyl)amino-N-(4-chlorophenyl)-3-methyl-4-isothiazolecarboxamide" [Supplementary Concept])	
13	**MeSH – "inflammatory cells":** "Cyclic Nucleotide Phosphodiesterases, Type 4"[Mesh] OR "Nedocromil"[Mesh] OR "Lymphomatoid Papulosis"[Mesh] OR "Interleukin-8"[Mesh] OR "Prostatitis"[Mesh] OR "Nephritis, Interstitial"[Mesh] OR "Granuloma, Plasma Cell"[Mesh] OR "Fasciitis"[Mesh] OR "Chorioretinitis"[Mesh] OR "Histiocytoma, Angiomatoid Fibrous" [Supplementary Concept] OR "GGTI-2133" [Supplementary Concept] OR "Distal myopathy, Nonaka type" [Supplementary Concept]	**12**
14	**[tw] – text words:** Antiinfecti* [tw] OR antimicrobial [tw] OR "antimicrobial activity" [tw] OR antibacterial [tw] OR anti-fungal [tw]	**208784**
15	**MeSH – Antimicrobial:** ((("Anti-Infective Agents"[Mesh] AND "Anti-Infective Agents" [Pharmacological Action] AND "Anti-Infective Agents, Local"[Mesh] AND "Anti-Infective Agents, Urinary"[Mesh] AND "Antimicrobial Stewardship"[Mesh] AND "Disk Diffusion Antimicrobial Tests"[Mesh] AND "Antimicrobial Cationic Peptides"[Mesh] AND "antimicrobial peptide, Pharbitis" [Supplementary Concept] AND "Anti-Infective Agents, Local" [Pharmacological Action] AND "Fatty Acid Synthase, Type II"[Mesh] AND "Fatty Acid Synthesis Inhibitors"[Mesh] AND "Oxyquinoline"[Mesh] AND "Sulfathiazoles"[Mesh]) AND ("Microbial Sensitivity Tests"[Mesh] OR "Immunity, Innate"[Mesh] OR "Hydroxyquinolines"[Mesh] OR "Drug Resistance, Microbial"[Mesh] OR "antimicrobial peptide 2, Lippia sidoides" [Supplementary Concept] OR "antimicrobial peptide 1, Lippia sidoides" [Supplementary Concept] OR "antimicrobial peptide AG-30" [Supplementary Concept] OR "antimicrobial peptide ESF39A" [Supplementary Concept] OR "antimicrobial hybrid peptide CM15" [Supplementary Concept] OR "antimicrobial peptide V4" [Supplementary Concept] OR "dehydrobatzelladine C" [Supplementary Concept] OR "P18 antimicrobial peptide" [Supplementary Concept] OR "SC5 synthetic antimicrobial peptide" [Supplementary Concept])) AND ("Fleroxacin"[Mesh] OR "Pefloxacin"[Mesh] OR "Enoxacin"[Mesh] OR "Norfloxacin"[Mesh] OR "Trimethoprim"[Mesh] OR	**85**

	"Infection"[Mesh] OR "antimicrobial peptide-P5" [Supplementary Concept])) AND "7-dibromo-2-methyl-8-benzoyloxyquinoline" [Supplementary Concept]	
16	**MeSH – antibacterial:** "Anti-Bacterial Agents"[Mesh] OR "Anti-Bacterial Agents" [Pharmacological Action] OR "Protein Synthesis Inhibitors"[Mesh] OR "Microbial Sensitivity Tests"[Mesh] OR "lacticin NK34, Lactococcus lactis" [Supplementary Concept] OR "N-acetylmuraminidase protein, Entamoeba histolytica" [Supplementary Concept] OR "CAP18 lipopolysaccharide-binding protein" [Supplementary Concept] OR "Tazobactam"[Mesh] OR "2-sulfoether-4-quinolone" [Supplementary Concept] OR "Bacillus clausii"[Mesh] OR "Drug Resistance, Multiple, Bacterial"[Mesh] OR "Drug Resistance, Bacterial"[Mesh] OR "antibacterial polypeptide LCI" [Supplementary Concept] OR "CAP11 protein, Cavia" [Supplementary Concept] OR "Hp (2-20) protein, Helicobacter pylori" [Supplementary Concept] OR "cecropin B3 protein, insect" [Supplementary Concept] OR "cecropin B1 protein, insect" [Supplementary Concept] OR "DEFB4A protein, human" [Supplementary Concept] OR "ASABF protein, Ascaris suum" [Supplementary Concept] OR "PMAP-37" [Supplementary Concept] OR "PMAP-36" [Supplementary Concept] OR "porcine myeloid antibacterial peptide 23" [Supplementary Concept] OR "defensin protein, Aeshna cyanea" [Supplementary Concept] OR "attacin antibacterial protein, insect" [Supplementary Concept] OR "Antimicrobial Stewardship"[Mesh]	**28**
17	**MeSH – antifungal:** "Antifungal Agents"[Mesh] AND "Antifungal Agents" [Pharmacological Action] AND "Drug Resistance, Fungal"[Mesh] AND “14-alpha Demethylase Inhibitors"[Mesh]	**10**
18	**[tw] – text words:** “Wound healing” [tw] OR “wound contraction” [tw] OR “cell proliferation” [tw] OR “Cell migration” [tw] OR wound [tw] OR lymphoedema [tw] OR lymphedema [tw]	**435,997**
19	**MeSH: Wounds-** ("Negative-Pressure Wound Therapy"[Mesh] AND "Wound Infection"[Mesh] AND "Wound Healing"[Mesh] AND "Re-Epithelialization"[Mesh] AND "Wounds, Stab"[Mesh] AND "Wounds, Penetrating"[Mesh] AND "Wounds, Nonpenetrating"[Mesh] AND "Multiple Trauma"[Mesh] AND "CT-102 activated platelet supernatant" [Supplementary Concept] AND "WTV S12 protein, Wound tumor virus" [Supplementary Concept] AND "Degloving Injuries"[Mesh]) AND ("Naa35 protein, rat" [Supplementary Concept] OR "WIND1 protein, Arabidopsis" [Supplementary Concept] OR "FGFR1OP2 protein, mouse" [Supplementary Concept] OR "wound-inducible transcript 3.0, rat" [Supplementary Concept] OR "wound-inducible ribonuclease, Nicotiana" [Supplementary Concept] OR "WIPK	**34**

	protein, Nicotiana tabacum" [Supplementary Concept] OR "SNK 863" [Supplementary Concept] OR "WIP1 protein, Zea mays" [Supplementary Concept] OR "Win-2 protein, Solanum tuberosum" [Supplementary Concept] OR "aloe vera gel extract hydrogel dressing" [Supplementary Concept] OR "Win-1 proteins, plant" [Supplementary Concept] OR "acemannan" [Supplementary Concept] OR "Wound tumor virus" [Supplementary Concept])	
20	**MeSH: Lymphedema-**<br>"Lymphedema"[Mesh] OR "Non-Filarial Lymphedema"[Mesh] OR "Yellow Nail Syndrome"[Mesh] OR "Hypotrichosis-Lymphedema-Telangiectasia Syndrome" [Supplementary Concept] OR "Lymphedema of the lower extremities, recurrent pneumonia, bronchiectasis, and yellowed nails" [Supplementary Concept] OR “Stewart Treves syndrome" [Supplementary Concept]	**21**
21	**[tw] – text words:**<br>Ethiopi* OR endemi* [tw]	**103231**
22	**MeSH – Ethiopi*:**<br>("Ethiopia"[Mesh]) AND (“Africa, Eastern"[Mesh] OR "filifolone" [Supplementary Concept] OR “isogeranic acid" [Supplementary Concept])	**17**
24	1 OR 2 OR 3 OR 4	**2,037,201**
25	5 OR 6	2
26	7 OR 8 OR 9 OR 10	**62355**
27	11 OR 12 OR 13	**648829**
28	14 OR 15 OR 16 OR 17	**868894**
29	18 OR 19 OR 20	**439629**
30	21 OR 22	**103231**
31	24 OR 25 AND 26 OR 27 OR 28 OR 29 AND 31	**9881**
29	Limit to English AND other animals AND (abstract OR Free full text OR Full text)	**1824**
